# Prediction of lisinopril pediatric dose from the reference adult dose by employing a physiologically based pharmacokinetic model

**DOI:** 10.1186/s40360-020-00429-y

**Published:** 2020-07-29

**Authors:** Memoona Rashid, Muhammad Sarfraz, Mosab Arafat, Amjad Hussain, Nasir Abbas, Muhammad Waqas Sadiq, Muhammad Fawad Rasool, Nadeem Irfan Bukhari

**Affiliations:** 1 Punjab University College of Pharmacy, University of the Punjab, Lahore, Pakistan; 2 Faculty of Pharmacy and Pharmaceutical Sciences, University of Alberta, Edmonton, Canada; 3 College of Pharmacy, Al Ain University, Al Ain, Abu Dhabi, UAE; 4 Clinical Pharmacology & Quantitative Pharmacology, Clinical Pharmacology & Safety Sciences, R&D, AstraZeneca, Gothenburg, Sweden; 5 Bahauddin Zakariya University, Multan, Pakistan

**Keywords:** Lisinopril, PBPK, Pediatric, PK-Sim MoBI®, Area under the curve

## Abstract

**Background:**

This study aimed to assess the pediatric lisinopril doses using an adult physiological based pharmacokinetic (PBPK) model. As the empirical rules of dose calculation cannot calculate gender-specific pediatric doses and ignores the age-related physiological differences.

**Methods:**

A PBPK model of lisinopril for the healthy adult population was developed for oral (fed and fasting) and IV administration using PK-Sim MoBI® and was scaled down to a virtual pediatric population for prediction of lisinopril doses in neonates to infants, infants to toddler, children at pre-school age, children at school age and the adolescents. The pharmacokinetic parameters were predicted for the above groups at decremental doses of 20 mg, 10 mg, 5 mg, 2.5 mg, and 1.5 mg in order to accomplish doses producing the pharmacokinetic parameters, similar (or comparable) to that of the adult population. The above simulated pediatric doses were compared to the doses computed using the conventional four methods, such as Young’s rule, Clark’s rule, and weight-based and body surface area-based equations and the dose reported in different studies.

**Results:**

Though the doses predicted for all subpopulations of children were comparable to those calculated by Young’s rule, yet the conventional methods overestimated the pediatric doses when compared to the respective PBPK-predicted doses. The findings of previous real time pharmacokinetic studies in pediatric patients supported the present simulated dose.

**Conclusion:**

Thus, PBPK seems to have predictability potential for pediatric dose since it takes into consideration the physiological changes related to age and gender.

## Background

Administration of a right dose is a critical factor to obtain optimum systemic drug concentration and its therapeutic effect. Any deviation from the optimum systemic drug concentration may lead to toxic or sub-therapeutic drug levels [1]. According to the guidelines of FDA, EMA and equivalent drug regulatory authorities, effective and safe dose and dose adjustments are needed in different clinical situations and for the other covariates, i.e., age, gender, obesity, pregnancy and disease states, i.e., renal disease [2] or hepatic disease [3].

The efficacy, safety and tolerability of different dosage forms of a drug are determined usually in adult population during phase I clinical trials [4]. Due to ethical, technical and regulatory restrictions, pharmacokinetic and clinical studies in the pediatric population are scarce [5]. Thus, pediatric dose is calculated from that of adult using the empirical formulae [6, 7] based on the size, age, weight and body surface area (BSA) of pediatric patients. Nevertheless, the above conventional methods have certain limitations; the Young’s rule (Eq. 1) ignores the varied sizes, weights and genetics of the individuals of the same age [8]. The Clark’s rule (Eq. 2) considers a linear relationship, between dose and weight, which indeed is otherwise. Computation of BSA-based (Eq. 3) pediatric dose is considered relatively reliable among the other methods for dose calculation [4, 9, 10].

1 $$ \mathrm{Youn}{\mathrm{g}}^{\prime}\mathrm{s}\ \mathrm{Rule}=\frac{\mathrm{Age}}{\left(\mathrm{Age}+12\right)} $$

2 $$ {\mathrm{Clark}}^{\prime}\mathrm{s}\ \mathrm{Rule}=\frac{\mathrm{Weight}\ \left(\mathrm{lb}\right)}{150}\times 100 $$

3 $$ \mathrm{BSA}\ \left({\mathrm{m}}^2\right)=\frac{\sqrt{\mathrm{Height}\ \left(\mathrm{Cm}\right)\times \mathrm{Weight}\ \left(\mathrm{Kg}\right)}}{3600} $$

The conventional methods are assumptions-based and ignore all the age-dependent physiological difference which can affect the pharmacokinetics and pharmacodynamics of a drug [11]. For instance, the absorption and metabolizing capacity, expression of enzymes in the liver, parameters relevant to distribution (such as protein binding), and excretion process (such as glomerular filtration) differ in children and adults [12, 13]. Furthermore, existence of the physiological differences among the children of different age groups [1] warrants more reliable and accurate options for dose computation in children.

The European Union and FDA have enforced and encouraged the need of drug development study of a new chemical entity for pediatrics use by employing PBPK model approach [14] for being a more advanced and sophisticated tool for the prediction of pharmacokinetic parameters in individuals of different age groups by considering the age-led physiological changes [15–19]. However, the PBPK approach is not without limitations, one of these is lacking literature to develop an accurate PBPK model and high technical expertise are required for correct model development.

In the recent years, the prevalence of primary hypertension in the pediatric population has increased largely. Its occurrence is about 3 to 5% in the USA and can be higher in other populations such as non-Hispanic blacks and Mexican Americans [20]. The model drug, lisinopril is being used for the treatment of hypertension in the pediatric population [21]. Limited dose-response and pharmacokinetic studies of lisinopril in pediatric populations had concluded varied pediatric doses [22–24]. Hence, there was a need for well-designed and established clinical studies of lisinopril in the pediatric population [25]. Lisinopril is an oral long-acting synthetic peptidyl dipeptidase inhibitor of angiotensin-converting enzyme (ACE) [26, 27], belongs to BCS III drugs, i.e., it has high water solubility and low permeability [28], and exhibits poor bioavailability (25%) with 6–60% inter-individual variability [29]. This inter-individual variability has been reported at all doses ranging from 5 to 50 mg of the drug [27]. Nevertheless, lisinopril has simple pharmacokinetics, excreted by renal route [30], does not undergo metabolism [25] and has saturable binding to ACE but does not bind with any other binding protein [31]. This drug has proven safety and effectiveness in adults, and as stated earlier, it is being used in children for hypertension. There was a need for developing a model which could predict lisinopril concentrations in various pediatric age groups to help in developing standard treatment guidelines.

To demonstrate the applicability of PBPK modeling for prediction of pediatric dose, we developed a PBPK model for lisinopril in adult population to scale down its dose to different subgroups of the pediatric population, by graded decremental dose method. To our best knowledge, we used a novel PBPK method for the prediction of pediatric dose. Previously, dose calculation using PBPK model involved the assessment of pharmacokinetics based on the dose range reported already from a clinical study [14] or accomplishing pharmacokinetics based on the dose computed from conventional methods, i.e., BSA- or weight-based equations [32, 33].

## Methods

### Software tool

PK-Sim-MoBI® (Version 5.4.3/3.4.3, Bayer Technology Services, Leverkusen, Germany; http://www.systems-biology.com), was used for the development of PBPK model. The age-dependent anatomical and physiological parameters of humans and different species used in the experimental studies have been in-built in the database of the tool [34].

### Development of adult IV PBPK models of lisinopril

**Fig. 1:**
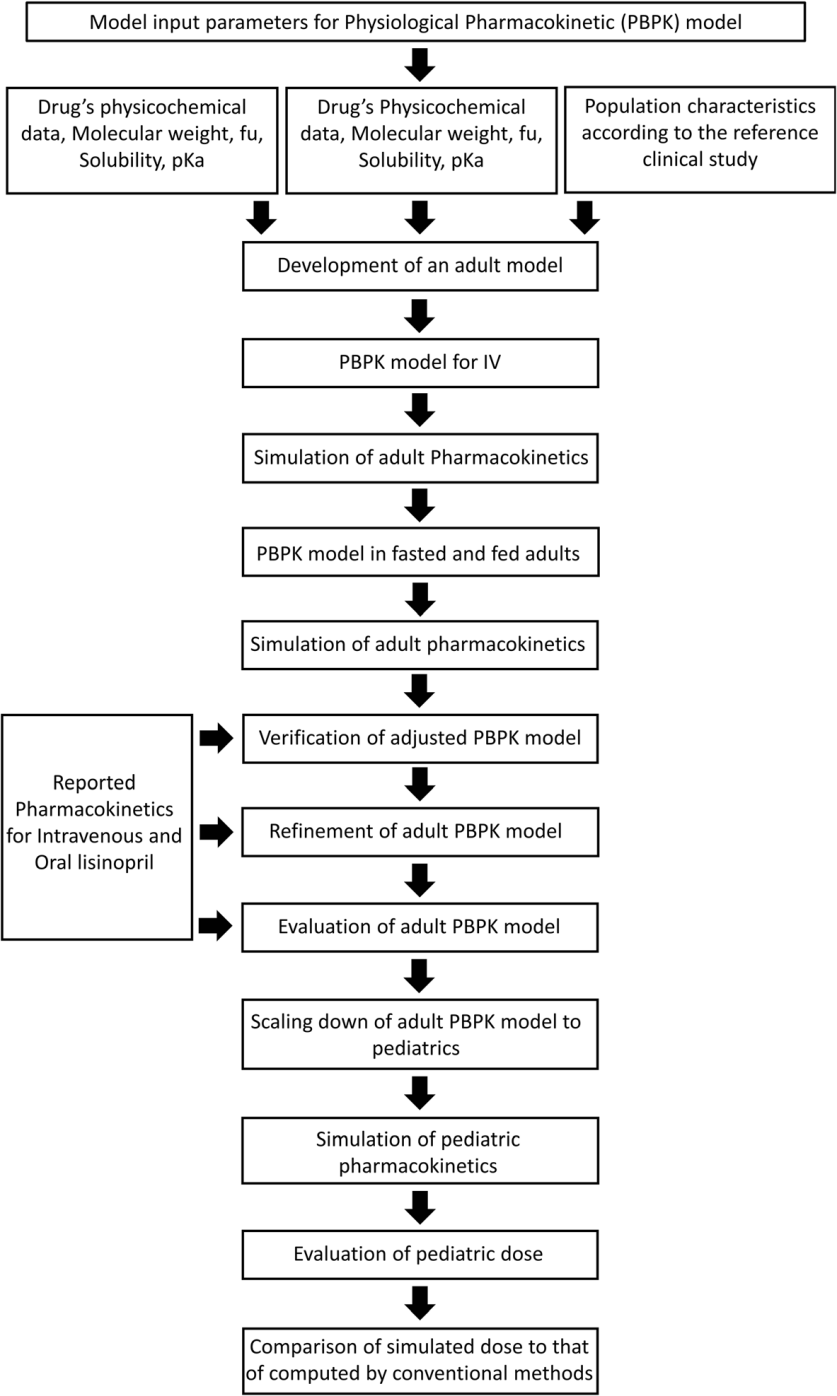
Developmental strategy for lisinopril PBPK model

A previously reported systematic approach [14, 35] was adopted for the development and validation of adult PBPK model of lisinopril (Fig. 1). For the development of PBPK model, all the population and drug specific parameters, including that of physicochemical and absorption, distribution metabolism and excretion (ADME), shown in Table 1 and Table 2 were entered as inputs under the respective nodes of PK-Sim.

**Table 1:** Physiological and biological input parameters for PK-Sim Simulation

Parameters/Properties		Values used	Reference values	References
**Physicochemical**	Lipophilicity (Log P)	-1.22	-1.22	[36]
	Plasma protein binding	Does not bind		[37]
	Molecular weight	405.48g/mol		[38]
	Pka	2.5	2.5 (at pH 7)	[36]
	Solubility (mg/L) at reference pH	97000	97000	[39].
**Biological**	Renal clearance	0.56 ml/min/kg	47±8.3ml/min 2.82±0.0083L/h	[40]
	Metabolism	-	No metabolism	[30]
	Elimination	Renal route	Renal route	[30]

**Table 2:** Characteristics of the healthy population used for lisinopril PBPK model development

Population Ethnicity	Population Size (n)	Proportion of female	Age (years)	Dose (mg)	Application	References
European	12	0	21–34	2.97	Single IV bolus	[41]
				5.53		
				11.20		
Caucasians	18	0	21–37	20	Single oral dose	[42]
European	28	0	18–42	20	Single oral dose	[26]
European	8	0	22–31	20	Single oral dose for 10 days	[41]
Neonates to Infants	100	50	0.25–1.0	5	Single oral dose	Current study
				2.5		
				1.5		
Neonates to toddler	100	50	0.25–1.9	20	Single oral dose	
				10		
				5		
				2.5		
				1.5		
Pre-schools children	100	50	2–5	20	Single oral dose	
				10		
				5		
				2.5		
				1.5		
School children	100	50	5–12	20	Single oral dose	
				10		
				5		
				2.5		
				1.5		
Adolescent	100	50	12–16	20	Single oral dose	
				10		

### Development of adult oral PBPK models of lisinopril

The values of different model input parameters required for the development of PBPK models of lisinopril is given as:

- Age: A clinical study on lisinopril in adult population of age range 21 to 37 [42] was selected as reference. According to the reference clinical study, a mean virtual healthy adult individual of age 29 (mean of 21 and 37) years was created for PBPK modeling of 20 mg lisinopril.
- Population ethnicity, age, weight and height: Population characteristics such as ethnicity, age, weight, and height of the entire population were created according to the values given in the reference study [42]. The PK-Sim-default values for ideal body weight (IBW) and heights for reference age range (21 to 37) were used during creation of population. A standard deviation of ±10% was entered for the values of heights.
- Expression level of transporter: Lisinopril is a substrate for the efflux peptide transporter-1, which is maximally expressed in duodenum and jejunum and less in ileum and colon present at the brush border of the small intestine [43]. Taking the above into account, for the creation of population, the expressed level of the transporter was taken as 100% in the PK-Sim data base.
- Input physiological and biological parameters: The required input physiochemical and biological parameters of lisinopril are given in Table 1.
- Dose of lisinopril: In line with the reference clinical study of lisinopril [42], a single dose tablet of 20 mg lisinopril was set as the administration protocol and the pediatric dose was determined by graded decremental doses to obtain a range of optimized dose.
- Meal calories for fed state: For the fed state, an event of 524 kcal meal was set according to reference [42]. Based on the above protocols, simulations for fasting and fed states were created for the whole population.
- Release pattern: In the formulation building block of PK-Sim, lint type release was entered as the type of drug release. The lint type release, the built- in node of PK-Sim also requires the time interval, as input at which a dosage form releases 80% of the drug. For this purpose, a compendial standard time of 30 min for 80% lisinopril release from the tablet was used as input [44].
- Intestinal permeability: The PK-Sim default value for intestinal permeability was adjusted manually in order to obtained same fraction of drug absorbed, peak blood concentration, bioavailability and area under the curve. The manual adjustment of intestinal permeability has also been supported in the literature to obtain matched pharmacokinetic parameters with the reference [30]. The adjustment of intestinal permeability for the prediction of the desired fraction absorbed in this study was in compliance with the previous report [14]. The adjusted value of human intestinal permeability was used in all simulations for adult and pediatric populations.
- Gastric emptying time: The default mean values of gastric emptying time and small intestine transit time were used in the prediction of the pharmacokinetics of each virtual population.
- Renal clearance: The optimized value of renal clearance was obtained through parameter identification function of PK-Sim for simulated pharmacokinetic profiles of lisinopril after an oral dose in fed and fasted states. Finally, the developed adult PBPK model was validated by comparing the predicted pharmacokinetic parameters to the reference pharmacokinetic parameters.

### Validation of PBPK models for adult IV and oral lisinopril pharmacokinetics

For validation of the PBPK model, the predicted pharmacokinetic parameters for three different intravenous bolus doses and multiple dosing were compared to those of the reported clinical study [41]. Similarly, the pharmacokinetic parameters of 20 mg lisinopril in fed and fasting states were compared to the reported pharmacokinetic parameters [26, 41, 42]. This validated PBPK model was used for development of the pediatric PBPK model for computation of the pediatric dose.

### Development of pediatric PBPK model

For the development of pediatric oral PBPK model for lisinopril, the validated adult PBPK model was scaled down to the pediatric populations of age 0–16 years [14]. The pediatric population was grouped, according to the standard age into, neonates to infants, infants to toddler, children of pre-school age, children of school age and adolescents by taking into the account of the age-related physiological changes in children. Each virtual pediatric group consisted each of 50 females and males. During creation of virtual pediatric population and simulation of PBPK model, physiological information related to this age group (0–16 years), including blood flow to different organs, GIT radius, length and effective surface area were scaled by PK-Sim itself according to the age. Other scalable parameters, gastric emptying time, intestinal transit time, and gastric pH, were set at the default values.

The demographic characteristics of the virtual pediatric population used for the model development are given in Table 2**.** The pharmacokinetics parameters of a single 20 mg adult dose of lisinopril were simulated in pediatric group, i.e., II (Infants to toddler), III (pre-school age children), IV (school age children), and V (adolescent) based on age. As expected, the values of PK parameters at the adult dose were higher. Thus, the PK parameters were re-calculated by decreasing the doses from 20 mg to 10 mg, 5 mg, 2.5 mg and 1.5 mg which corresponded to a dose reduction of 50, 75, 87.5 and 92.5%, respectively to accomplish the age-specific desired PK profiles and parameters in Group II, III, IV and V. The PK profile and parameters of lisinopril for neonates to infants (group I) were calculated at doses of 5, 2.5, 1.5 and 1 mg.

## Results

### Simulated pharmacokinetic profiles of lisinopril after doses and protocols

**Fig. 2:**
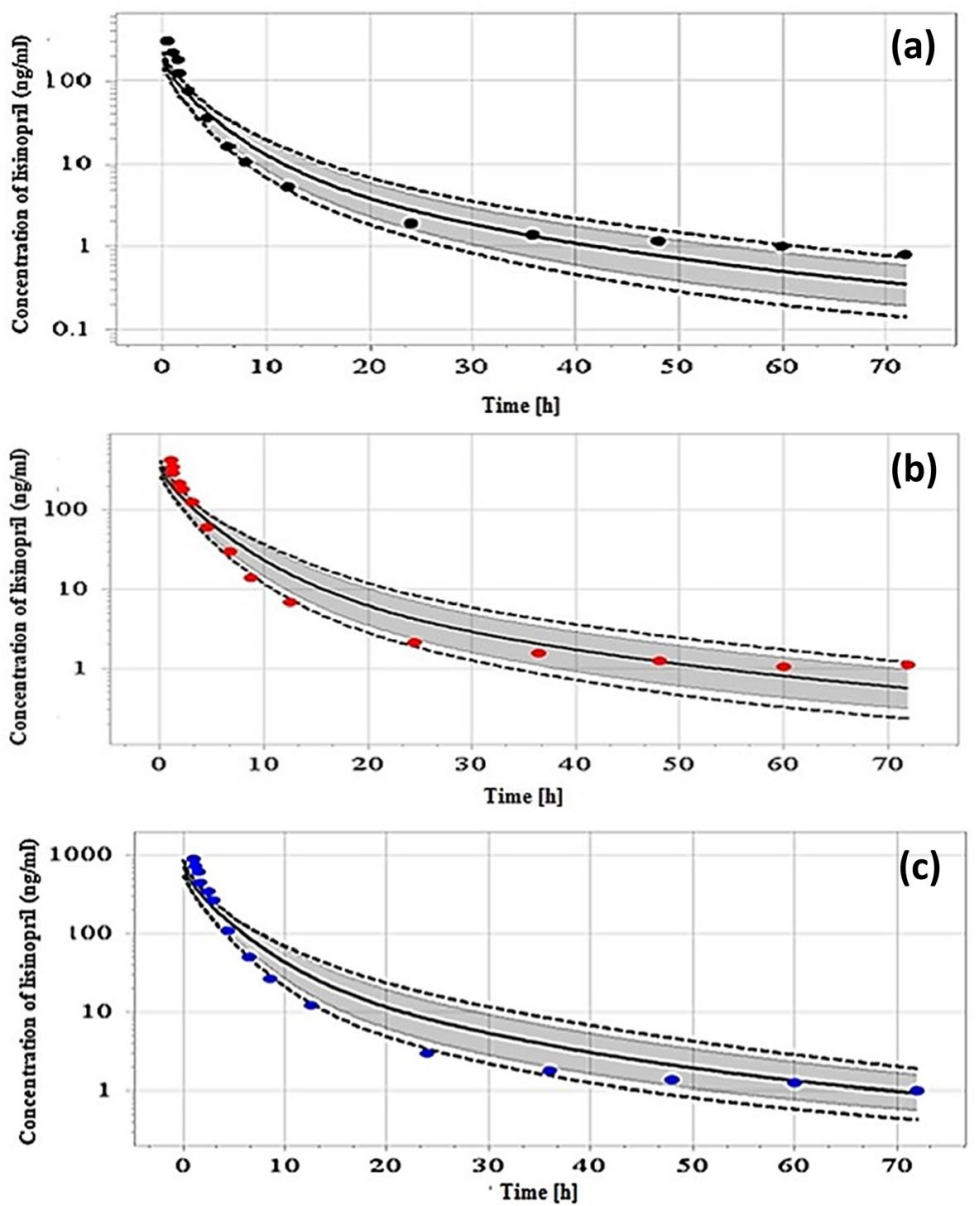
Predicted plasma concentration time profile shown as solid line and observed data of IV bolus doses: **a** 2.97 mg as black dots, **b** 5.53 mg as red dots and **c** 11.20 mg as blue dots. Dotted lines show minimum and maximum value and shaded area shows 5th and 95th percentile

**Fig. 3:**
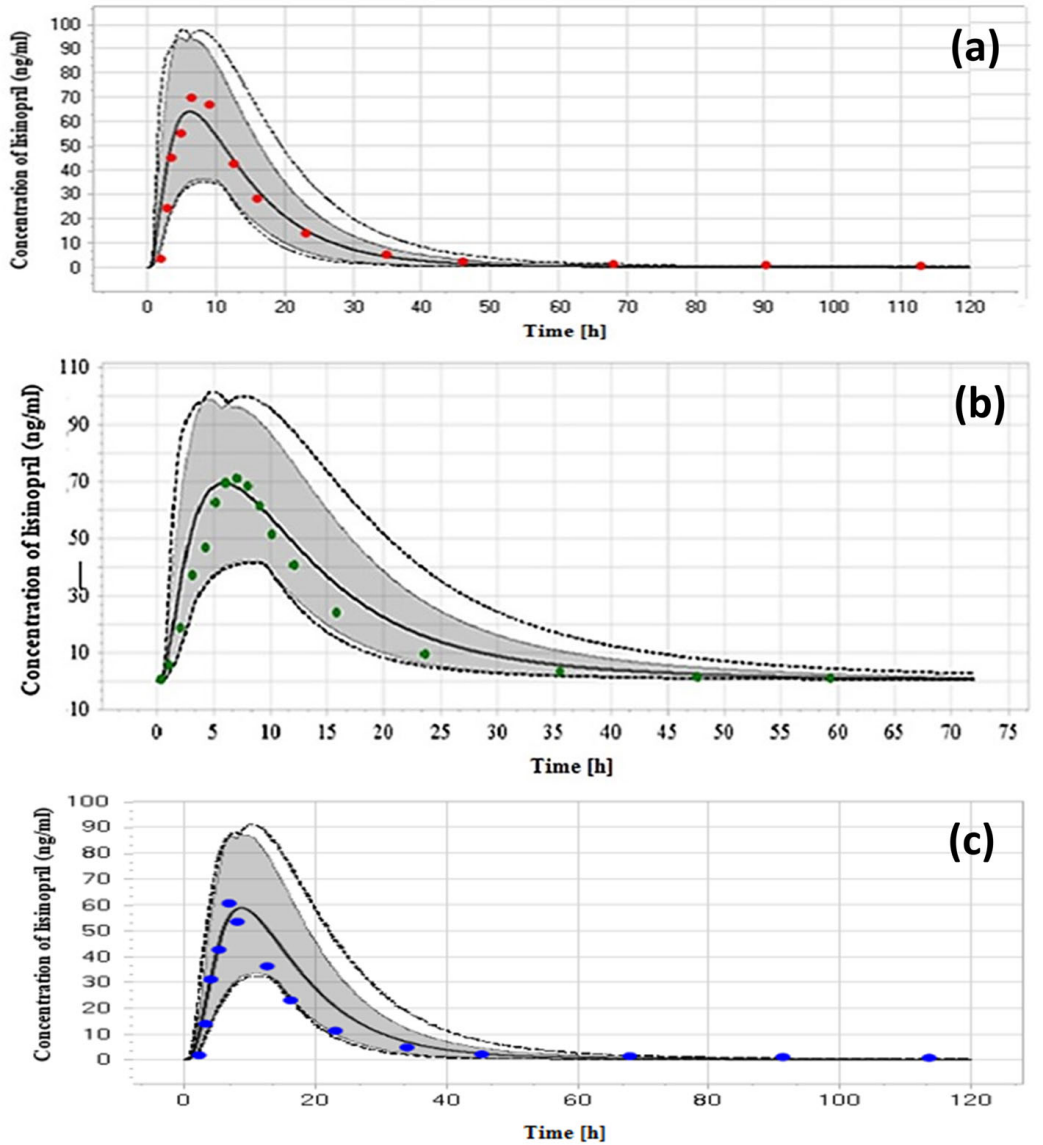
Predicted plasma concentration-time profiles of lisinopril shown as solid line and observed data in fasting as red and green dots (**a)** and (**b**), and fed state as blue dots (**c**) after 20 mg oral single lisinopril dose in healthy adults according to reported clinical studies [26, 30]. Dotted lines show minimum and maximum values while shaded area shows 5th and 95th percentiles

**Fig. 4:**
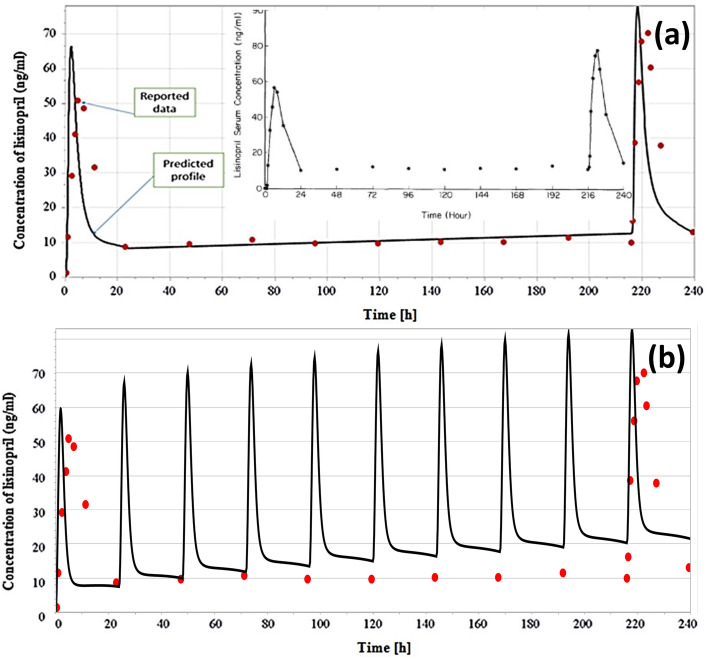
Predicted plasma concentration profile of lisinopril shown as black line and observed data as red dots after 10 multiple oral doses, (q24 h): **a** based on reference study [41], given in inset after only 1st and 10th doses and, **b** complete profile after 10 daily doses, predicted as black line and observed as red dot

Simulated plasma concentration profiles compared to the reported profiles and pharmacokinetic parameters for the three different single IV doses in this study are shown Fig. 2 and Table 3, respectively. For fasting condition, the predicted plasma concentration profiles in Caucasian adult healthy male volunteers of age 21–37 (mean = 29) years, after 20 mg of lisinopril single dose, are shown in Fig. 3a and b while for the fed condition, these are shown in Fig. 3c. Table 3 shows the predicted peak lisinopril concentration (C_max_), time reaching to C_max_ (T_max_) and the area under the curve from time zero to the last time point (AUC_0–120h_) in fasting and fed states after oral 20 mg lisinopril. The simulated plasma level time profiles and parameters were compared to that of the reference study [42]. The simulated profile for the multiple dosing of lisinopril 20 mg OD tablet given for 10 days is given in Fig. 4. Predicted mean minimum plasma concentrations (C_min_) of lisinopril after 1, 2, 3, 4, 5, 6, 7, 8, 9 and 10 days of multiple dosing were 8.40, 11.45, 12.20, 12.40, 12.48, 12.53, 12.57, 12.60, 12.63 and 12.79 ng/ml, respectively. The predicted C_min_ values were compared to values reported in the reference [41]. The C_min_ is the drug concentration just prior to the administration of next dose and exhibits, along with other factors the drug equilibration with tissues [45]. We reported C_min_ because this parameter was referred in the reference study [41], generally more frequently reported, and considered to be less varied, accurate, and reliable [45].

**Table 3:** Predicted pharmacokinetic parameters of lisinopril after IV and oral dose administration

Ethnicity	Protocol	Dose OD (mg)	C_**max**_ (ng/ml)		Tmax (h)		AUC_**0-t**_ (ng.h/ml)		References
			Observed	Predicted (Mean)	Observed	Predicted (Mean)	Observed	Predicted Min-Max	
European	IV dose	2.97	–	182.3	–	0.1	682 ± 156^**a**^	403–848.9	[41]
		5.53	–	347.2	–	0.1	1026 ± 123^**a**^	753.9–1598	
		11.2		707		0.1	1884 ± 107^**a**^	1453–3100	
Caucasians	Oral (Fasting)	20	86 ± 48	65.52	6.2 ± 1.1	6.15	1231 ± 620^**b**^	495–2097.6	[42]
	Oral (Fed)	20	69 ± 19	58.9	6.8 ± 1	7.6	1029 ± 254^**b**^	525–2124.7	
European	Oral (Fasting)	20	79.8 ± 39.4	69.32	6.5 ± 1.7	5.6	992.8 ± 520^**a**^	556.7–2298	[26]

AUC_0-t_^a^: Area under the curve from time zero to 72 h

AUC_0-t_^b^: Area under the curve from time zero to 120 h

### Simulated pharmacokinetics profiles in healthy pediatric population

**Fig. 5:**
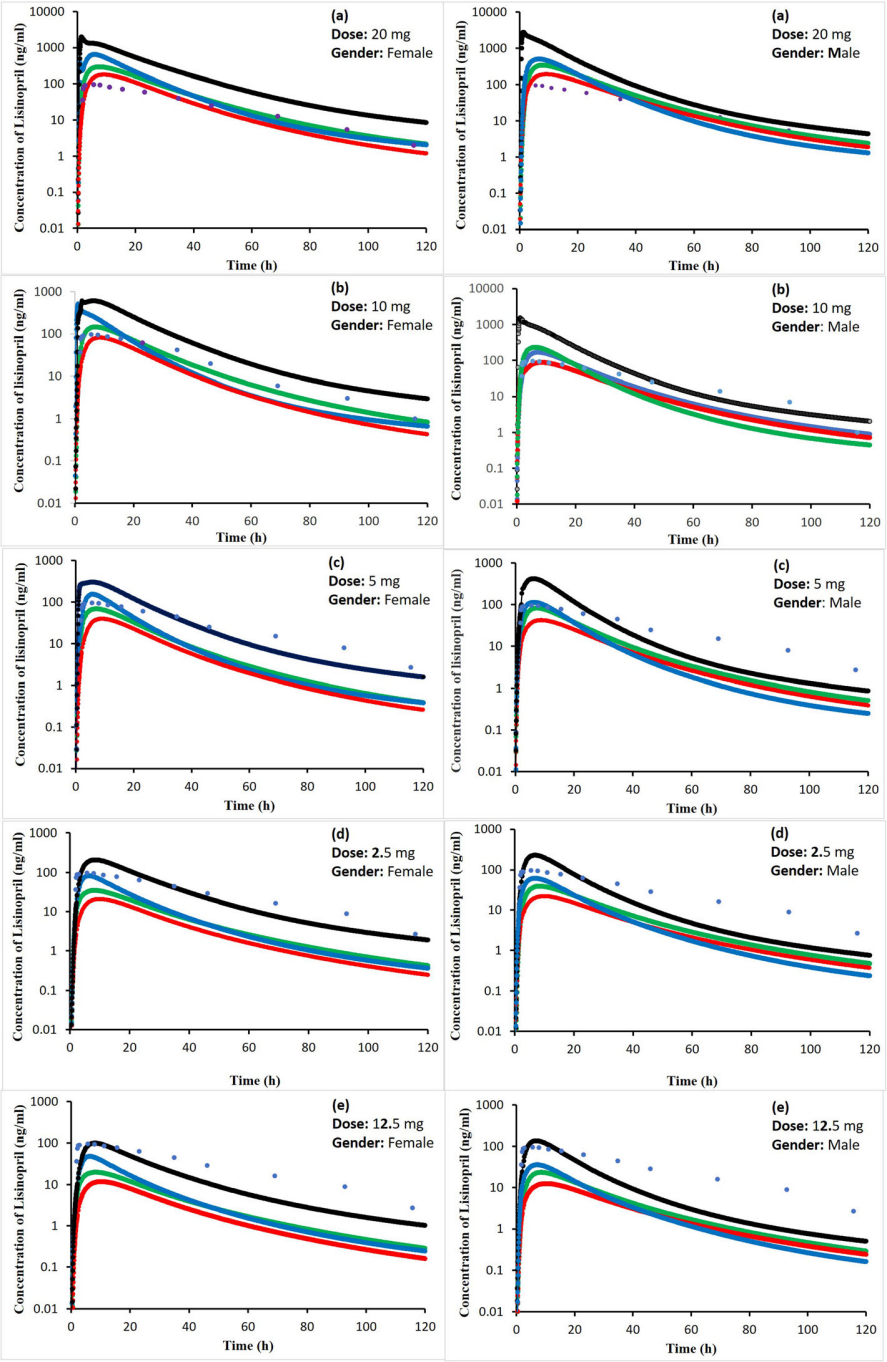
Simulated lisinopril plasma concentration profiles after administration of: (**a**) 20 mg (**b**), 10 mg (**c**), 5 mg (**d**), 2.5 mg and, (**e**) 1.5 mg for both genders in infants to toddler as black line, pre-schooled children as blue line, schooled children as green line, adolescent as red line and observed experimental data in healthy adults as dots

**Fig. 6:**
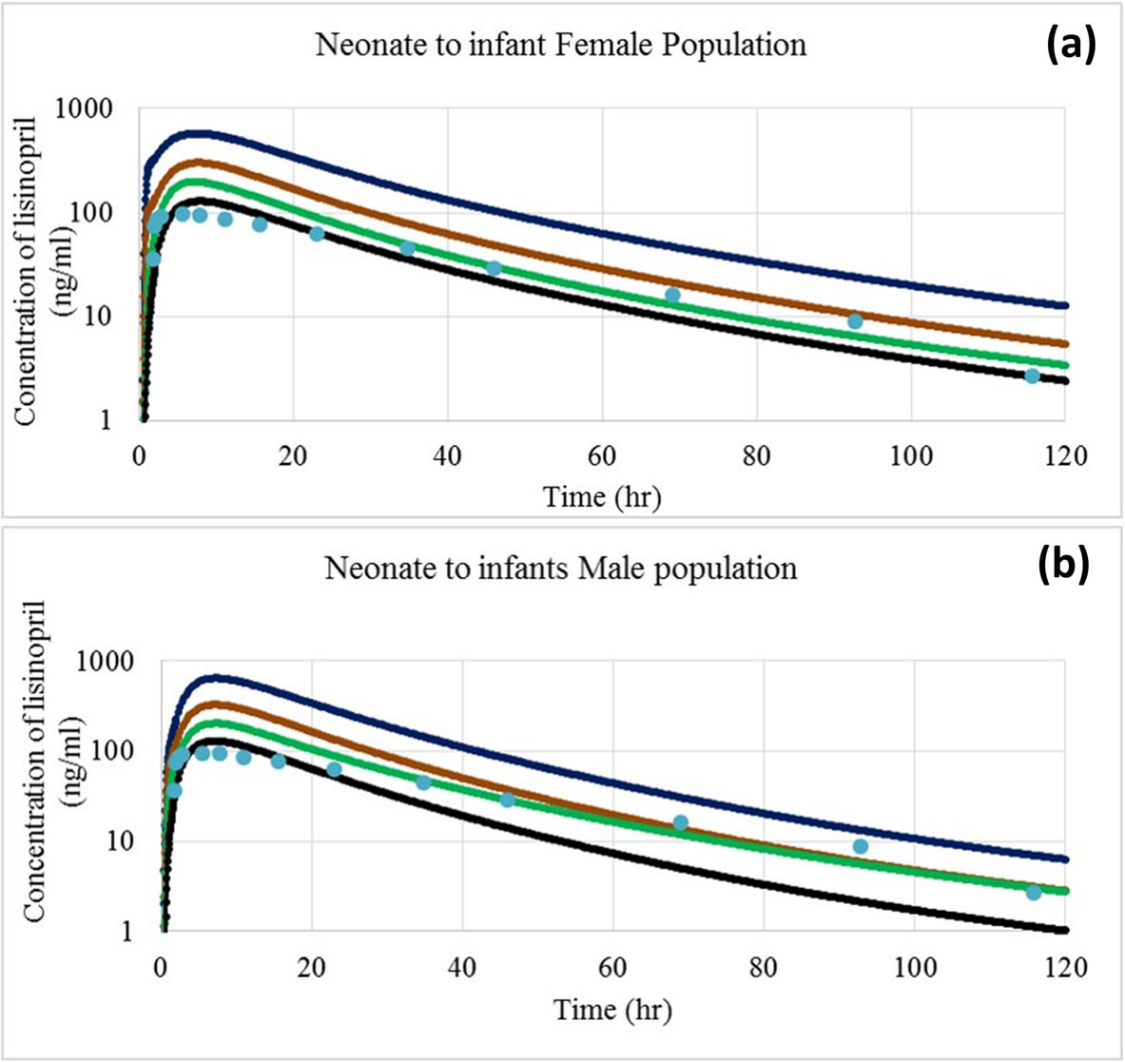
Simulated plasma concentration profiles of lisinopril in neonates to infants: **a** female and **b** male at different doses, 5 mg as blue line, 2.5 mg as brown line, 1.5 mg as green line, and 1 mg as black line and observed experimental data in healthy adults as dots

The trigger to start dose from 20 mg of lisinopril was a previous clinical study [42], which was used as reference for adult model development. From this adult dose, the pediatric dose was determined by graded decremental doses of 50, 75, 87.5 and 92.5% in order to obtain therapeutic doses. The predicted doses for specific age group were compared to that of the doses calculated using conventional methods (Table 5) and those reported in previous studies. The plasma concentration profile of lisinopril for pediatric population simulated at adult dose (20 mg) and after graded dose reduction of 50, 75, 87.5 and 92.5% of the adult dose (i.e., 10 mg, 5 mg, 2.5 mg and 1.5 mg) is shown in Fig. 5 a, b, c, d and e, respectively. The simulated C_max_, T_max_ and AUC_0–120h_ have been given in Table 4. In neonates to infants, the simulated plasma concentration profile and AUC_0–120h_ after doses of 5 mg, 2.5 mg, 1.5 mg and 1 mg are shown in Fig. 6 and Table 4.

**Table 4:**
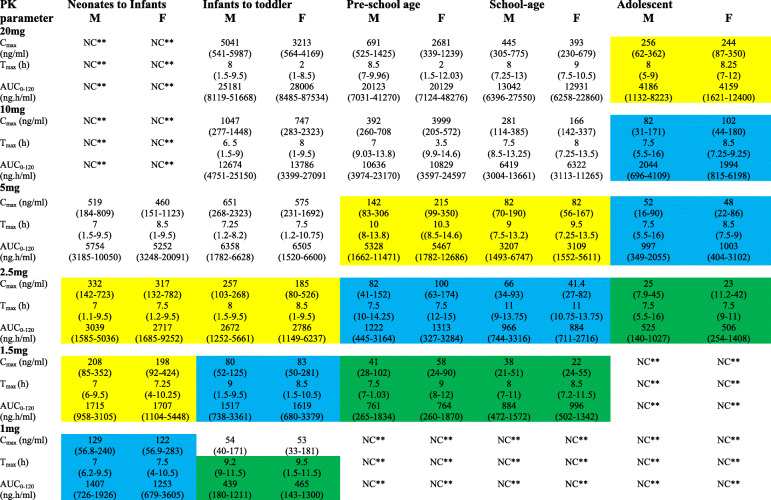
Predicted pharmacokinetic parameters of lisinopril after different single oral dose administration to pediatric population

* [42], NC** Not Calculated

The pharmacokinetic parameters were predicted at different doses to obtain values in pediatrics, similar to the reference adult values predicted at 20 mg, such as C_max_ 86 ± 48 (range 38–124) ng/ml, T_max_ 6.2 ± 1.1 (range 5.1–7.3) h and AUC_0–120_ 1231 ± 620 (range 611–1851) ng.h/ml. The yellow color indicates falling of lower range of predicted C_max_ and AUC_0–120_ within the range of reference values, Blue color shows falling of lower and mean value of predicted AUC_0–120_ in reference range, while green color demonstrates dose at which maximum value of C_max_ and AUC_0–120_ falls in reference range

## Discussion

### Reasons for selection of lisinopril

Lisinopril follows simple pharmacokinetics, shows no protein binding [31], does not undergo metabolism [25] and is excreted unchanged through renal route [30]. The GFR of such drugs is not affected by age, as it reaches to above 90% of adult level at the age of 1 year [46], thus clearance is also unaffected with age [47]. The above features of the drug could help easy PBPK model development and reliable validation of the simulated findings. Furthermore, findings of a real-time studies in hypertensive and kidney transplant pediatric recipients for their pharmacokinetics parameters [48] are available, which could be matched with the simulated values in the present study for authenticated simulation by PBPK modeling.

### Simulated pharmacokinetic profiles of lisinopril after three different single IV bolus doses

The predicted plasma concentration time profiles and the pharmacokinetic parameters for three IV doses were compared to the values reported in a study taken as reference [41]. In the reference study, the plasma level time profiles (Fig. 2) were predicted after three intravenous bolus doses (2.97, 5.53, and 11.20 mg) for 12 European healthy male adults, age 21 to 34 year. The pharmacokinetics simulation with IV application provides its disposition kinetics without the interference of complexities arising from absorption [14]. The predicted plasma concentration profiles (Fig. 2) and pharmacokinetic parameters (Table 3) for the three IV doses were comparable to the reference study [41]. The predicted value of V_ss_ in this study after IV protocol was comparable to its reported value (i.e., 0.89 L/Kg) in the reference study [49]. All model input parameters which resulted into the similar visual depiction of pharmacokinetic profiles and parameters to that of the above reference study along with the other parameters influencing absorption (i.e., intestinal permeability, gastric emptying time and intestinal transit time) were employed for the simulation after oral dose in the next step.

### Simulated pharmacokinetics of lisinopril after an oral dose in fed and fasted states

Input of the model-produced value of intestinal permeability, 1.89E^− 9^ cm/min, resulted 25% lesser bioavailability for lisinopril than reported bioavailability (with 6–60% inter individual variability) [29]. Literature supports redefining of parameters, particularly value of intestinal permeability in order to accomplish similar-to-desired pharmacokinetic profile [14, 50]. Thus, intestinal permeability was adjusted from range of permeability values entered in parameters identification option of PK-Sim. As a result, permeability of 3.6E^− 7^ cm/min minimized variation between the fitted and reference bioavailability profiles [42]. Lisinopril shows a large inter-individual variability in a population after equal doses, study reports [51]. In the present study, bioavailability of lisinopril remained unaffected in presence of food, in line with previous report [37].

Two previous studies on lisinopril in fasting state [26, 30] could be used for authentication and validation of developed PBPK model in this study. Since both studies produced comparable pharmacokinetic parameters after same lisinopril dose in fasting adults therefore, for further prediction of pediatric dose, only one study [30] was employed as reference. The simulated C_max_, T_max_ and AUC_0–120h_ of lisinopril after oral 20 mg lisinopril were comparable to values reported by Beermann 1988. The predicted C_max_ in fasting and fed conditions (88.52 ng/ml and 61.36 ng/ml, respectively) were comparable to the reported values (86 ± 48 ng/ml and 69 ± 19 ng/ml) in same conditions [30]. Similarly, the predicted T_max_ values in fasting and fed conditions (6.15 h and 7.6 h, respectively), were also found comparable (Fig. 3 and Table 3).

### Simulated pharmacokinetic profile after multiple oral dosing of lisinopril

Multiple dosing of lisinopril 20 mg OD tablet for 10 days generated comparable plasma level profile (Fig. 4) and minimum plasma concentration (C_min_) to the reference [41].

### Predicted lisinopril pediatric dose

In neonates to infants and in infants to toddlers, the desired PK parameters were obtained at population dose of 1.0 and 1.5 to 2.5 mg shown in Table 4 and Figs. 5 and 6, respectively, while for the pre-school and school children, the predicted dose were 1.5, 2.5 and 5 mg by considering the mean, minimum and maximum value of C_max_, T_max_ and AUC_0–120h_. In adolescents, the predicted doses were 5 and 10 mg. The simulated Plasma time profiles (Figs. 5 and 6) and doses for all groups of children were compared to the doses calculated by Young’s rule (age), Clark’s rule (weight), and on weight (mg per kg) basis and according to the BSA of child using 20 mg as adult reference dose (Table 5). These formulae for calculation of a child dose are based on physiological covariates including age, weight, height or BSA.

**Table 5:** Doses calculated for children of different ages by using empirical formulas of pediatric dose calculation

	Pediatric dose calculation methods								PK-Sim Dose^**a**^ (mg)
Pediatric Population	Young’s rule		Clark’s rule		Weight (mg/kg)-based		BSA-based		
	Age (years)	Dose (mg)	Weight (pound)	Dose (mg)	Weight (kg)	Dose (mg)	BSA (m^**2**^)	Dose (mg)	
Infants to toddler	1	1.53	22	2.93	10	2.85	0.43	4.73	1.0 to 2.5
Preschool	5	5.88	37	5	17	5	0.78	8.5	1.5 to 5
School age	12	10	88	12	40	11	1.25	13	1.5 to 5
Adolescent	17	12	132	18	60	17	1.58	17	2.5 to 10

^a^Same doses were calculated for male and female children

The modelled pediatric dose for infant (1 year), 1.5–2.5 mg was similar to that found by Young’s and Clark’s formula. For the pre-school, 5-year old child, Young, Clark, weight-based and BSA-based formulae overestimated dose as 5.88, 5.0, 5.0 and 8.5 mg, respectively as compared to a remarkably lesser predicted dose of 2.5 mg. Similarly, in 12-year old child the doses given by Young and Clark, weight-based and BSA-based formulae, respectively were 10, 12, 11 and 13 mg, higher as compared to the model-computed dose, 1.5, 2.5 and 5 mg. The PBPK model proposed therapeutic dose range of lisinopril in the pediatric population of age 5 to 16 years as 1.5 mg to 10 mg.

Previous reports supported the present findings. According to the literature the dose of lisinopril in children of age 6 to 16 years is to be started from the 2.5 mg, which should then be increased gradually to achieve the antihypertensive effect [52]. A study in hypertensive children of age 7–17 years with stable kidney function following transplant concluded that lisinopril follows linear response on increasing the dose [24]. The lisinopril should be started from a low dose, i.e., 0.1 mg/kg which could be adjusted according to the response of patients. Our doses in the healthy pediatric population (Table 4) were comparable to the above findings. While FDA has approved the use of lisinopril for hypertensive children above 6 year and or who receive the kidney transplant with a starting dose from 5 mg to maximum dose of 40 mg [22]. Another study in hypertensive pediatric patients suggested 2.5 mg once daily dose of lisinopril in children of less than 6 year age with body weight less than 25 kg and 5 mg as dose for age greater than 6 years and body weight 25 to 45 kg. For children of age greater than 6 years and weight greater than 45 kg, the suggested dose was 10 mg [53] which was in line with the present findings (Table 4). The present dose was also supported by another study where lisinopril showed maximum antihypertensive efficacy at dose of 5 mg in children of 6–16 years age [23].

Mean simulated T_max_ of lisinopril for neonates to infants was, respectively 6 and 4 h in females and males, which was also comparable to that reported for children [25]. Dose predicted with PBPK model for an infant (1 year), preschool (5 years) and school (12 years) child were comparable to dose calculated by Young’s rule. While dose calculated by Clark’s rule, weight (mg/kg) based equation and BSA-based formula were higher for infants, toddler, pre-school age, school age and adolescent population as compared to respective dose obtained through PBPK modeling. As there is 6 to 60% of variability for lisinopril at all reported dose ranges of 5-50 mg, therefore initially, a lower dose must always be started. Another limitation of the above formulae is their inability to compute dose separately, for male or female’s child coupled with ignorance of the physiological differences in different age groups. However, requirement of adjustments of intestinal permeability for prediction of pharmacokinetic parameters during simulation necessitated the validation of the current PBPK modeling. Nonetheless, the simulated findings seemed to be reliable as were supported by the following: (A) consistent findings of present simulations in healthy adults and with a study in hypertensive and kidney transplant pediatric recipients [48], (B) comparable simulated pharmacokinetic parameters in adults to that of reported after lisinopril administered to different pediatric subpopulations in doses 0.1–0.2 mg/kg (C) existence of a strong correlation of GFR-dependent drug clearance to BSA and, as a result comparable PBPK model predicted and BSA-based doses of lisinopril as GFR remains unaffected with age, since it reaches > 90% of adult levels by age 1 [54], and lisinopril clearance remains unaffected with age, as lisinopril is not metabolized, and excreted largely unchanged through GFR, and (D) demonstration of drug as effective, safe and well tolerated when dosed according to recommendations in previous investigations consistent with the findings of this study. Dose computation though PBPK approach could be reliable if extensive adult pharmacokinetic data is available. Furthermore, the effective application of the PBPK for the dose calculation in pediatric patients requires dose confirmation in the real clinical setting.

## Conclusion

The PBPK models using dose decremental method could be employed for the prediction of lisinopril pediatric dose, particularly by taking into consideration of the age-led changes in specific pediatric subpopulations. The PBPK approach is seemed to predict also the gender-specific doses which is not possible in conventional methods. Dose extrapolation in children solely based on age or weight may not be accurate and may potentially be harmful to children. PBPK approach may have more dose prediction potentials as it considers the physiological changes related to age. However, the finding of this study could be translatable clinically only after a real time pharmacokinetic or clinical study in such patients with the predicted doses.

## Data Availability

All the data analyzed or generated during this study is given in manuscript. Extra information can be obtained from corresponding author, if needed.

## Abbreviations

AUC: Area under curve
BSA: Body Surface Area
C_max_: Maximum plasma concentration
EMA: European medicine agency
FDA: Food and drug administration
IV: Intravenous
Min: Minimum
Max: Maximum
OD: Once daily
PBPK: Physiological Based Pharmacokinetic model
T_max_: Time to reach a maximum plasma concentration
